# The effect of empathy on remote social connections via paired robots

**DOI:** 10.1371/journal.pone.0316240

**Published:** 2025-01-30

**Authors:** Satoru Suzuki, Noriaki Imaoka, Takeshi Ando, Misako Uchida, Kenji Suzuki

**Affiliations:** 1 Robot Promotion Office, Manufacturing Innovation Division, Panasonic Holdings Corporation, Kadoma, Osaka, Japan; 2 Artificial Intelligence Laboratory, University of Tsukuba, Tsukuba, Ibaraki, Japan; Tongji University, CHINA

## Abstract

A range of devices and technologies are available to mediate social connections between geographically distant people. Some of these methods exploit awareness information to enhance the connectedness of distant users. However, the effect of user traits on the experience of interpersonal communication through awareness systems remains unclear. In this study, we explored the relationship between user empathy and the experience of interpersonal communication mediated by a paired robot able to convey user awareness information. We conducted an experiment to facilitate communication among distant family members using the paired robot over the course of 1 week. Participants’ empathy level and their experience of the communication were assessed using questionnaires. The findings indicated that individuals with higher empathy can better interpret their partner’s situation and feelings from the awareness information.

## Introduction

Staying connected with family members is important for the overall well-being of families [1, 2]. However, maintaining a sense of family connection can be challenging. This is particularly the case in Japan, where it is increasingly common for people to live in nuclear families and the proportion of parents and children living in separate households are growing. The annual increase in these types of family structures means that many families who are physically distant use communication technologies such as telephone, email, and chat apps to talk and exchange messages. These methods of communication tend to be used to convey specific information; therefore, people may feel reluctant to use them unless they have a specific purpose or need to contact others. The frequency of telecommunication is significantly influenced by lifestyle factors and geographical distance. Research indicates that weekly work hours and distance impact the contact frequency between parents and adult children [3]. Specifically, as weekly work hours increase and distance grows, the frequency of communication tends to decrease. To maintain family connectedness in separated living conditions, it is essential to address the communication challenges posed by the differing lifestyles of parents and adult children. This situation underscores the importance of exploring alternative methods that facilitate effective communication and sustain family bonds despite these obstacles.

Research aimed at supporting social connections among individuals in distant locations has primarily focused on using awareness of the activities and status of remote individuals. Various modalities, including light, sound, vibration, pressure and temperature, have been employed to convey awareness information through specialized devices [4–7]. Their effectiveness in fostering social connections has been demonstrated for separated individuals, such as family members [8, 9] and romantic partners [10, 11]. However, how user traits affect the experience of interpersonal communication through awareness systems remains unclear. It is important to understand the relationship between user traits and the experience of interpersonal communication to develop an effective awareness system.

Empathy plays an important role in interpersonal communication. Many studies have been conducted to investigate the importance and effects of empathy. In the healthcare setting, nurses rely on empathetic connections to establish rapport with patients and deliver affective care [12, 13]. Individuals with low empathy often encounter challenges in communication, as evidenced by the difficulties faced by individuals with Autism Spectrum Disorder (ASD) compared with their neurotypical counterparts [14, 15]. Therefore, the ability of empathy is essential for facilitating successful communication and may play a significant role in interpersonal communication facilitated by awareness systems.

The study objective was to comprehensively investigate the effect of empathy on communication mediated by a robot that can convey user’s awareness information. We believe that, since the amount of information conveyed by an awareness system is quite limited compared to usual communication methods, the ability to imagine other’s intentions and activities from this limited information is essential for better interpersonal communication. We hypothesized that the way the robot is used and how they experience communication through the robot would change depending on their empathy ability. To validate the hypothesis, we conducted an experiment on the experience of communication among distant family members using a robot that can convey user awareness information. Empathy level and the experience of interpersonal communication was evaluated using the Empathy Quotient (EQ) [16] and a questionnaire, respectively.

The contributions of this paper are as follows: (1) We have demonstrated that the level of empathy significantly influences communication when mediated by a robot capable of conveying users’ awareness information. (2) The findings indicate that individuals with higher empathy are better at interpreting the situations and emotions of their partners. (3) We have provided design implications of the robot that can enhance the quality of communication, particularly for users with lower empathy levels.

## Related work

Various approches have been proposed to support social connections between people in distant locations. Video-based technologies such as VideoWindow [17] and Portholes [18] use videos or still images to convey the states of remote users. VideoWindow displays a video of remote locations on a large screen and Porthole displays a list of still images of remote users every few seconds. Although these methods enable the spontaneous acquisition of information about remote individuals using videos and still images, they may pose substantial risk of violating the privacy of individuals.

Some researchers have successfully developed systems that balance the protection of individual privacy and transmission of awareness information by minimizing the amount of information conveyed [19–21]. The main approach involves detecting some of the activities or status of individuals in remote locations and presenting this information to the devices of other user. Gaver et al. [19] developed a touch-based input/output device called Light Touch for long-distance communication. When a user touches a device, a paired device owned by the other user lights up. Liu et al. [20] introduced a smartwatch app that allows individuals to share and view each other’s biosignals, such as heart rate. The app represents heart rate as mood, using different shapes, movements, and colors to enhance expressiveness and playfulness. Degraen et al. [21] developed a device called FamilyFlower, which is an artificial flower designed to facilitate connections between remote households. FamilyFlower detects various modalities such as human presence, movement, sound, and touch. In response to these stimuli, a paired device opens a flower bud, activates the stem, changes the color of the seeds, and dispenses a fragrance. These studies have demonstrated their effectiveness in fostering social connections through the transmission of a limited amount of information.

However, the relationship between user traits and the experience of interpersonal communication through awareness systems is still not well understood. Gaining insights into this relationships is essential for the development of an effective awareness system. Research on human-robot interactions has shown that human traits such as personality [22] and attachment style [23] affect acceptance of robots. For example, individuals prefer to interact with a robot that has the same personality as they do (e.g., introverted individuals prefer an introverted robot) [24]. Some studies have attempted to provide robots that can adaptively change their behavior to match users’ personalities by estimating user personality from verbal [25] and nonverbal [26] behavior and generating the speech and gestures of the robot accordingly [27]. The findings of these studies indicate the importance of understanding the effect of user traits on interpersonal communication using awareness systems. This would help in the development of effective awareness system to enhance long-distance social connections.

Empathy, which consists of cognitive and affective components, refers to the ability to comprehend the intentions, activities, and emotions of others while also sharing in their feelings [16]. This is an essential ability for facilitating smooth communication between individuals. Nevertheless, many studies have neglected to examine the influence of empathy when evaluating the effectiveness of awareness systems. Most research has focused on developing innovative interfaces, primarily focusing on their utility while overlooking the underlying factors that affect communication [28–30]. Among the existing literature, a few studies have explored the features of awareness systems that contribute to successful communication over long-term use exceeding two years [19], as well as the influence of participant relationships on their interactions [31]. Given this gap in the literature, it is essential to acknowledge that, although empathy is recognized as a key component of effective communication, its role has yet to be thoroughly examined in the context of awareness systems. In this study, we aim to highlight the importance of empathy and its potential impact on interpersonal communication when utilizing awareness systems. By doing so, we hope to contribute to a more nuanced understanding of how empathy can influence interpersonal communication within awareness systems.

## Materials and methods

We developed a prototype robot that can convey user awareness information with the objective of increasing social connections between parents and children in distant locations. The design of the robot, including physical form and its communication methods, was developed entirely by authors. The robot utilizes tactile switches to detect user touches, conveying this information to the other robot through physical changes, such as color shifts in its LED indicators and its hand motion. User empathy was assessed using the EQ and their experiences of the robot-mediated interpersonal communication was evaluated through a questionnaire. The relationship between user’s EQ and the responses to the questionnaire was statistically analyzed.

### Robot

**Fig 1:**
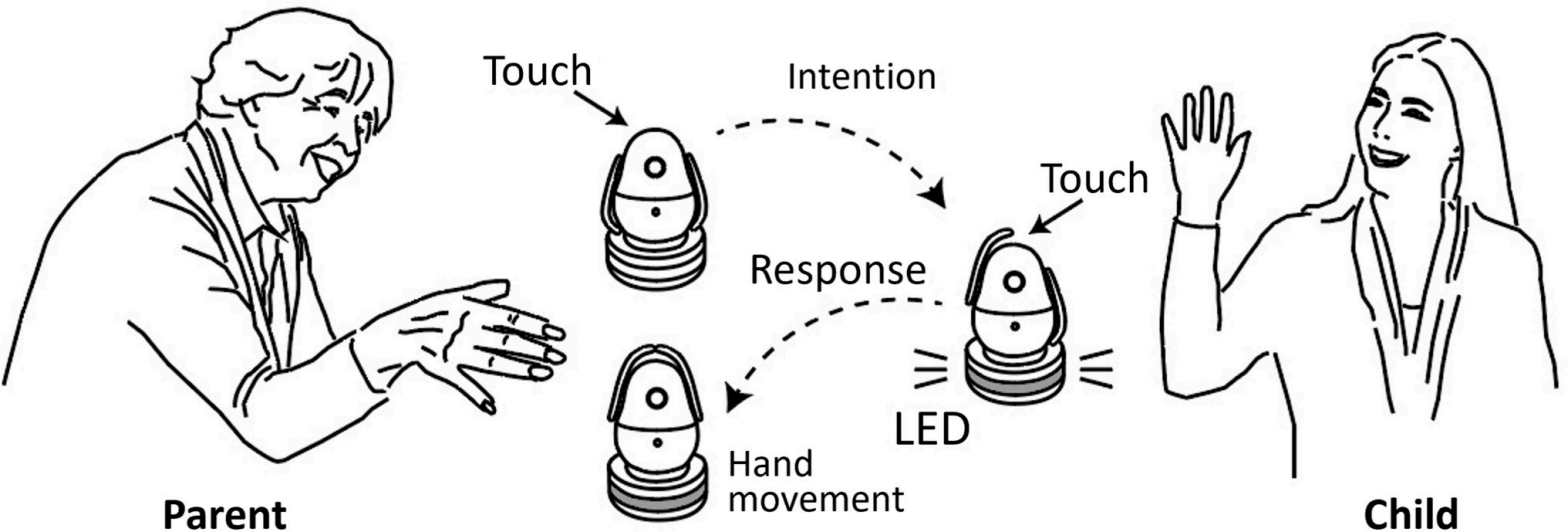
The interpersonal communication process using the robot.

Fig 1 illustrates the interpersonal communication process using the robot “cocoropa” developed for this study. A pair of robots were installed in each user’s home and both robots connected in a peer-to-peer manner through the Internet. Interaction via the robot is achieved simply by touching the robot’s head, and this information is conveyed to another user through the movements of the robot’s hands. When a user first touches the robot’s head, both robots raise their right hands. When the other user responds by touching the robot’s head, both robots raise their left hands. In this way, users can communicate with each other simply by touching the robot’s head.

**Fig 2:**
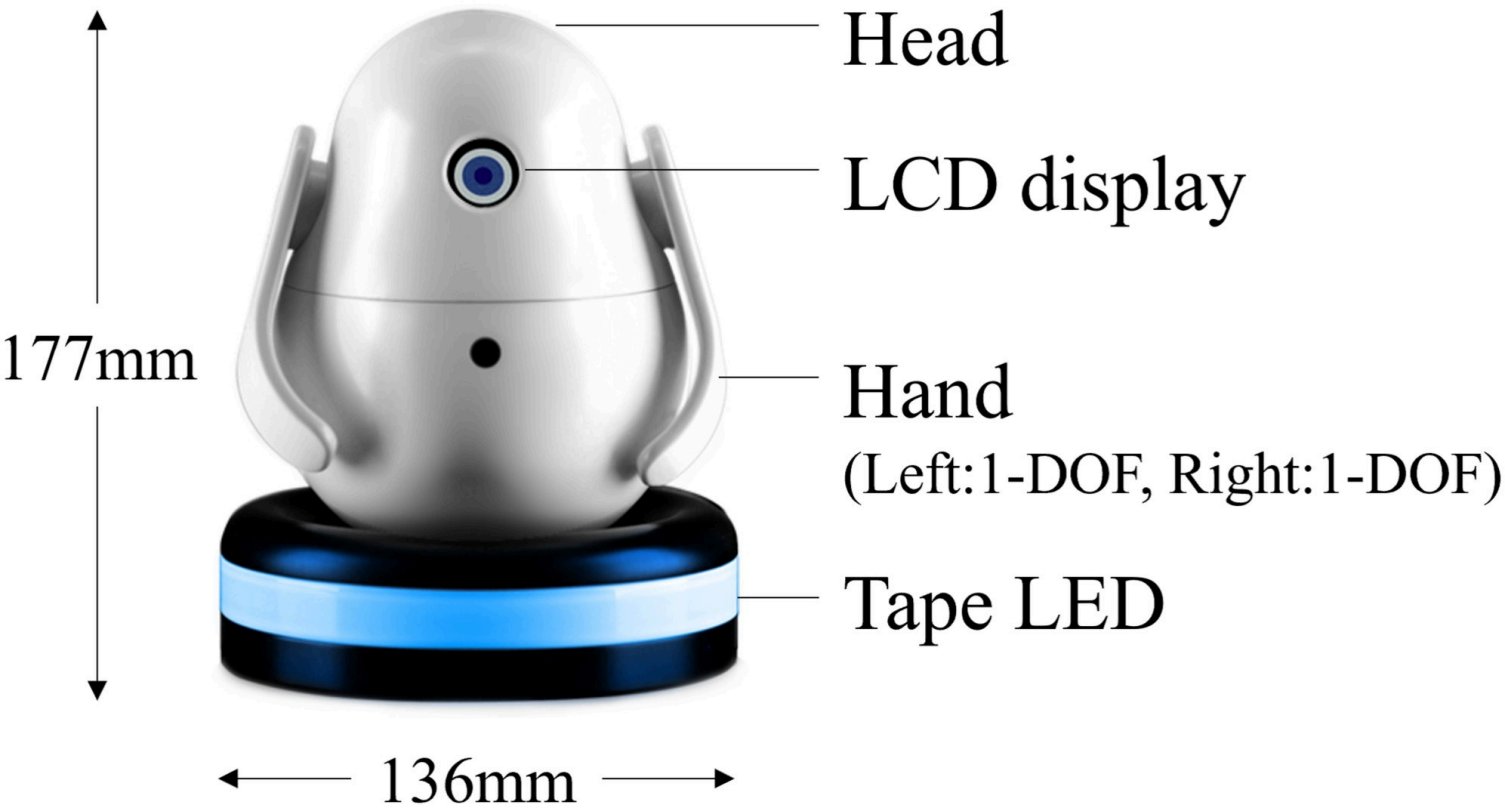
Robot configuration.

The robot device comprises the robot body and a pedestal (Fig 2). The robot body is equipped with a liquid–crystal display that represents the robot’s single eye, two servo motors that operate the robot’s two arms, and a microcomputer with a Wi–Fi module. The pedestal comprises a tape LED and four tactile switches. All modules are controlled by a microcomputer mounted on the robot’s body. Power is supplied by an alternating–current adapter; the power to the robot and pedestal is turned on by inserting a power plug into the insertion port of the pedestal. The robot has a width of 136 mm and a height of 177 mm when both hands are down, and a height of 193 mm when both hands are raised.

**Fig 3:**
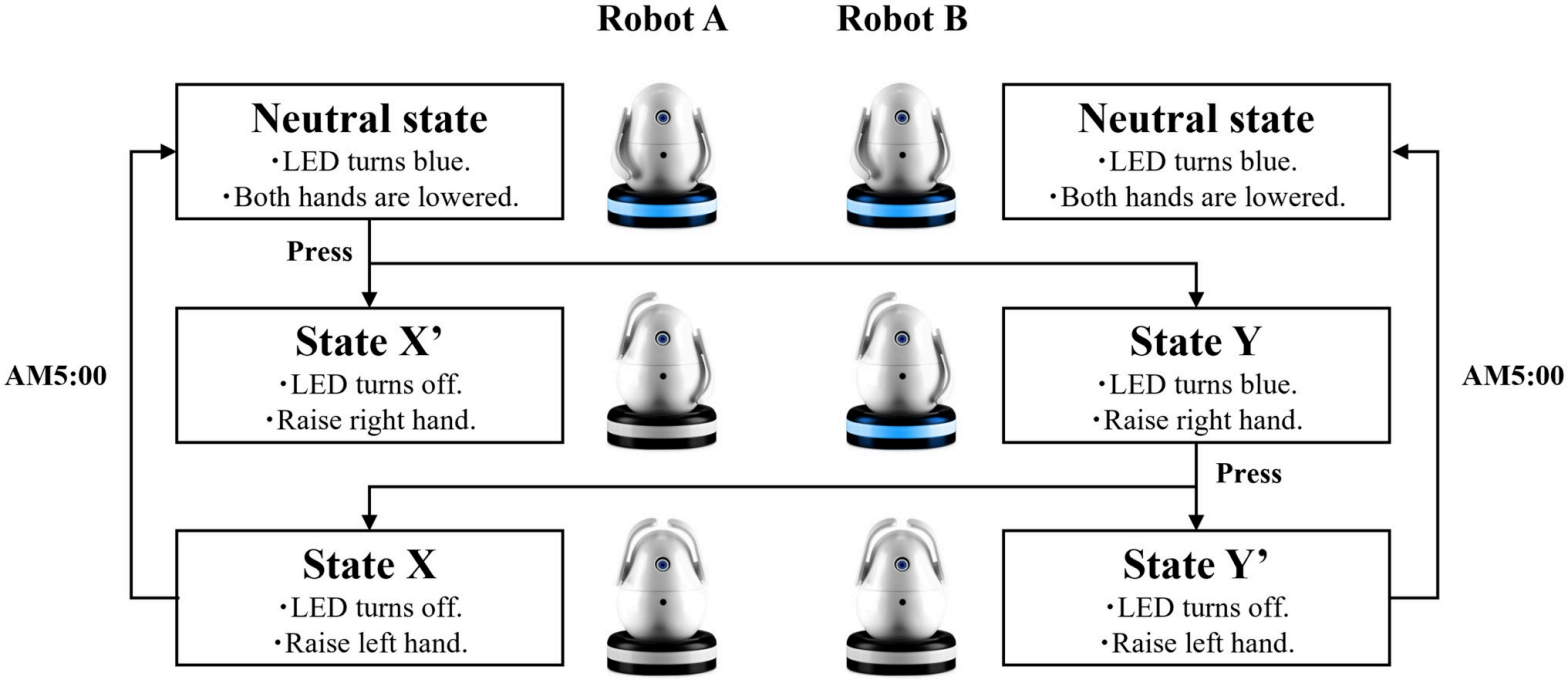
State transition diagram of paired robot.

Fig 3 is a state transition diagram of the paired robot. Each robot starts from a neutral state in which the LED on the pedestal lights up blue and both hands are lowered. When the head of robot A is touched, the right hand rises and the LED turns off. At the same time, the right hand of robot B rises and the LED remains blue. Then, when the head of robot B is touched, the left hand rises and the LED turns off. At the same time, the left hand of robot A also rises. Every morning at 5 a.m., both robots return to a neutral state in which their hands are lowered, and the LEDs light up blue. In this way, interaction using the robots is completed in one round trip per day. The order in which the robot heads are touched is arbitrary, so the head of robot A may be touched after the head of robot B is touched.

The LED color on the pedestal indicates the robot status, as shown in Table 1. The head of the robot can be touched when the LED is blue; touching the head when the LED is off or red produces no response. The hand of the robot represents the sending and receiving states, as shown in Table 2. The lowering of both hands indicates a neutral state, whereas the raising of the right hand indicates that a signal has been sent or received. The raising of both hands indicates that the heads of both robots have been touched and interaction has been completed for the day.

**Table 1:** LED color and robot status.

Color	Status
Blue	The head of the robot can be touched.
Red	Touching the robot’s head will produce no response owing to error.
Off	Touching the robot’s head will produce no response owing to the completion of its operation.

**Table 2:** Hand movements and robot states.

Hand movement	Status
Both hands lowered	Neutral state.
Right hand raised	A signal has been sent/received.
Both hands raised	Communication between the robots is completed.

### Empathy quotient(EQ)

We used the EQ [16] to measure participants empathy. The development of the EQ was based on the assumption that the affective and cognitive components of empathy co-occur and cannot be easily separated. Therefore, the EQ is an appropriate measure to comprehensively assess both cognitive and affective components of empathy. The validity and reliability of EQ have been demonstrated in [32, 33] and it has been utilized in a variety of studies to examine the effect of empathy on social support [34] and empathy levels in large-language models [35]. Based on these reasons, we adopted EQ as our measure of empathy.

The EQ questionnaire comprised 60 items, including 20 dummy items that were used to distract participants from the focus on empathy. For each item, participants were asked to select from four possible responses, ranging from ‘Strongly agree’ to ‘Strongly disagree’. The total EQ score comprised responses on 40 items (after excluding the 20 dummy items); the total possible EQ score range was 0 to 80. We used a version of the EQ questionnaire that we translated from English to Japanese.

To investigate the relationship between effects of EQ and the experience of our robot-based communication, we categorized individuals based on their EQ score. It is reported that people with low empathy often struggle with communication, as evidenced by comparative studies between individuals with ASD and their neurotypical counterparts [14, 15]. Insights from previous studies [16, 36] indicate that individuals with ASD and healthy individuals can be distinguished by an EQ score of 30. Based on these research findings, we believe that the experience of our robot-based communication significantly changes at an EQ score of 30. In this study, each participant was categorized into low-EQ and high-EQ groups using a threshold EQ score of 30. If both parent and child have higher EQ score, they are assigned to high-EQ group. But they can be assigned to different group if their EQ scores are low and high. For example, a parent with an EQ score over 30 is classified as high-EQ, while a child with an EQ score below 30 is classified as low-EQ group.

### User experience questionnaire

We developed a questionnaire to assess how communication was conducted using the robot and how effective this communication was. Table 3 shows the 26 questionnaire items, which were originally designed by the authors according to the characteristics of the robot and the nature of the experiment. The questionnaire consists of three sections assessing user attribute information, the experience of interpersonal communication using the robot, and the intended use of the robot.

**Table 3:** User experience questionnaire assessing user experience of robot-mediated communication.

No.	Question	*χ* ^2^	p–value
Q1	What is your age?	-	-
	(20s, 30s, 40s, 50s, 60s, ≥70s)		
Q2	What is your sex?	-	-
	(Male, Female, Other, No answer)		
	Please choose the answer that most closely describes your experience.		
Q3	Did you want to touch the robot (press the robot’s head) when you saw it?	3.10	0.21
	(Very much, A little, Not at all)		
Q4	Did you touch the robot for the purpose of communicating with the other person?	2.00	0.37
	(Often, Sometimes, Never)		
Q5	How did you feel about the other person when you pressed the robot’s head?	10.21^*1^	**0.00** ^*1^
	(Multiple selections allowed: Troubled, Worried, Angry, A sense of expectation, A sense of fun^*1^, No particular feeling, Other)		
Q6	Did you press the robot’s head because you enjoyed the movement and light when you pressed the head?	2.39	0.30
	(Often, Sometimes, Never)		
Q7	How did you feel when you were waiting for a reply from the other person?	10.21^*2^	**0.00** ^*2^
	(Multiple selections allowed: Frustrated, Worried, A sense of fun, A sense of expectation^*2^, No particular feeling, Other)		
Q8	Did you think of the other person when you received a reply from them?	9.48	**0.01**
	(Very much, A little, Not at all)		
Q9	How did you feel when you received a reply from the other person?	9.80^*3^	**0.00** ^*3^
	(Multiple selections allowed: Relieved, Worried, A sense of fun, Happy^*3^, No particular feeling, Other)		
Q10	Did you feel closer to the other person using the robot?	3.52	0.17
	(Very much, A little, Not at all)		
Q11	Did you feel like you were with the other person when using the robot?	1.19	0.55
	(Very much, A little, Not at all)		
Q12	Compared with before using the robot, did you think more about the other person?	8.09	**0.00**
	(Much more, A little more, Less)		
Q13	How did you feel about the other person?	N/A	N/A
	(Positive, Negative)		
Q14	How did you feel about interacting with the robot?	5.00^*4^	**0.03** ^*4^
	(Multiple selections allowed: Frustrated, Troubled, Relieved, A sense of accomplishment, A sense of fun^*4^, Happy^*5^, No particular feelings, Other)	5.95^*5^	**0.01** ^*5^
Q15	Did you get tired of using the robot?	6.85	**0.03**
	(Often, Neutral, Never)		
Q16	Did you have conversations with the other person about the robot?	0.60	0.74
	(Very much, A little, Not at all)		
Q17	Compared with before using the robot, did you have more opportunities to make contact by phone or e–mail or LINE (messaging app)?	0.00	0.93
	(More, Same, Less)		
Q18	Was it easy to detect the reply from the other person?	2.18	0.34
	(Very easy, Easy, Not easy)		
Q19	How satisfactory was the frequency of daily exchanges (one round trip)?	0.00	1.00
	(Just right, Too many, Too few)		
Q20	What frequency would be ideal?	-	-
	(Please give the number of times per day.)		
Q21	Do you feel that continuing to use the robot would help you maintain and deepen your relationship?	3.76	0.15
	(Very much, A little, Not at all)		
Q22	Do you want to continue using the robot?	0.08	0.78
	(Yes, No)		
	Please choose the answers that most closely match your intended uses.		
Q23	To tell the other person that I will do something or that I have done something.	0.08	0.78
	(Yes, No)		
Q24	To check whether the other person is ready to reply or to encourage the other person to reply.	0.21	0.65
	(Yes, No)		
Q25	To make the other person aware of a sent e–mail or incoming call, or to encourage the other person to access their mobile phone.	0.00	1.00
	(Yes, No)		
Q26	To encourage the other person to contact me by other means such as an e–mail or phone call.	0.04	0.83
	(Yes, No)		

### Statistical analysis

A t-test was used to investigate whether there are gender differences or parent-child differences in EQ. The average EQ scores for males and females, and for parents and children were calculated, and their differences were assessed statistically. To examine the associations between EQ score and the responses to the questionnaire, a chi-squared test was employed. A chi–square tests were performed on responses to all questions except Q1, Q2, and Q20 in Table 3. As questions Q5, Q7, Q9, and Q14 allowed multiple responses, a chi–square test was conducted on responses to each option. When statistically significant results were obtained in a chi-square test, residual analysis was applied to find which responses were associated with EQ score.

### Experimental conditions

The experiment was conducted with pairs of participants. We asked each participant to place a robot in their home at a location of their choosing. The number of times that the robot head could be touched was limited to once per day, so the communicative exchange was completed when each pair of participants touched the robot head once a day. We asked participants to touch the robot head at a time of their choosing. The robot status was reset at 5 a.m. every morning, so all new interactions began after 5 a.m. To ensure that only the study participants used the robot, participants were asked to ensure that their cohabitants did not touch the robot. During the experiment, there were no restrictions on participants communicating with each other via email or telephone; this ensured that the robots were used in a natural daily life setting.

### Experimental procedure

**Fig 4:**
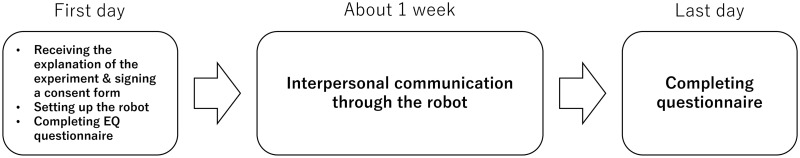
Experimental procedure.

The experimental procedure is summarized in Fig 4. Participants first received written and video explanations of the nature of experiment and signed a consent form for participation. Participants then set up the robot by themselves according to the user manual. The robot setup is very simple and requires connecting the robot and a mobile router to an electrical outlet. The robot can then send and receive signals to its paired robot via an Internet connection. After confirming that the robot was set up correctly, participants completed the EQ online. Following these preparations, participants started to communicate with their partners the next day. The experimental study period was approximately 1 week. After completing 1 week of communication, participants were asked to complete an online user experience questionnaire. Additionally, we conducted interviews with some participants using an online conference system (participants were asked for their consent to conduct the interviews).

### Participants

We recruited parent–child pairs who did not live together. A total of 10 parent-child pairs participated in the experiment. The parents (two men and eight women) were in their 50s to 70s and the children (four men and six women) were in their 30s to 50s. There were two experimental periods of approximately 1 week each: the first five parent-child pairs participated in the experiment from November 1st, 2021 to November 9, 2021, and the other pairs participated from November 29, 2021 to December 6, 2021.

The experiment was carried out with the approval of the ethics committee of the University of Tsukuba (2021R497).

## Results

### EQ score

The EQ scores of the 20 participants ranged between 22 and 48, with a mean score of 31.5. There were no significant differences in EQ scores between male and female (*t*(18) = 1.73, *p* = 0.051) or between parents and children (*t*(18) = 2.11, *p* = 0.096). Therefore, we analyzed the relationship between EQ scores and user experience questionnaire scores without considering other factors. Participants were categorized into low-EQ and high-EQ groups using a threshold EQ score of 30. There were 8 participants in the low–EQ group and 12 participants in the high–EQ group.

### User questionnaire results

**Fig 5:**
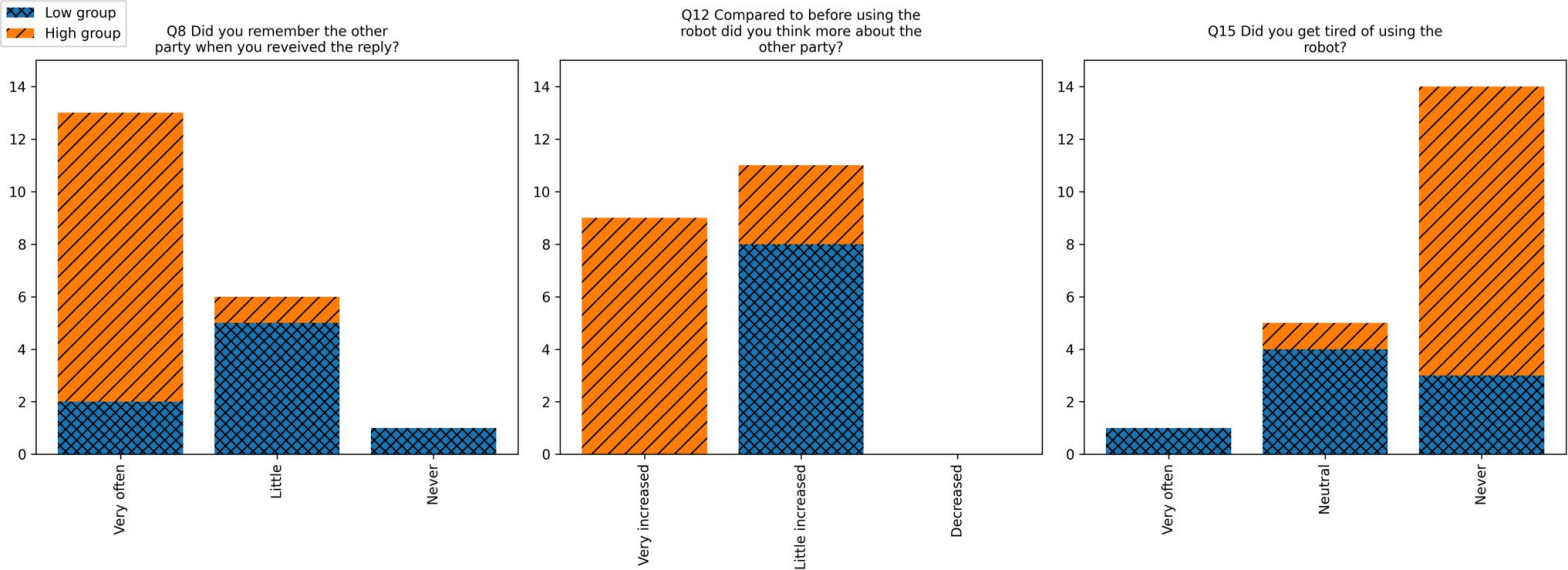
The responses to the questionnaire for low-EQ and high-EQ groups.

We anticipated that the experience of robot-mediated interpersonal communication would vary according to individual EQ score. To examine this, we conducted chi-square tests to assess the relationship between EQ scores and responses on the user experience questionnaire. *x*^2^ and *p*-value columns in Table 3 shows the results of the chi–square tests. Bold *p*–values represent statistically significant associations between EQ scores and the responses. For questions that allowed multiple responses, only the statistically significant results are shown with an asterisk (*). Statistically significant results were observed for Q8, Q12, and Q15. Fig 5 shows the responses on the user experience questionnaire for the low-EQ and high-EQ groups. A residual analysis was therefore conducted on the responses to these questions to determine which responses were associated with EQ score. Tables 4–6, respectively, show the results of the residual analysis for Q8, Q12, and Q15. Each column represents the number of the responses to the questionnaire and the standardized residual. Bold *p*–values represent statistically significant associations between EQ scores and each response. Table 4 shows that there were significant associations between EQ scores and the responses ‘Very much’ and ‘A little’; that is, individuals with higher EQ scores were more likely than those with lower EQ scores to think of the other person when they received a reply from that person. Table 5 shows that there were significant associations between EQ scores and the responses ‘Much more’ and ‘A little’, indicating that individuals with higher EQ scores were more likely than those with lower EQ scores to think more about the other person compared with before using the robot. Table 6 shows that there were significant associations between EQ scores and the responses ‘Neutral’ and ‘Never’, indicating that individuals with higher EQ scores were less likely than those with lower EQ scores to tire of using the robot. Table 3 also shows that there were significant associations between EQ scores and positive responses to Q5, Q7, Q9, and Q14. These results show that individuals with higher EQ scores experienced more positive emotions than those with lower EQ scores during the robot–mediated interaction.

**Table 4:** Residual analysis of ‘Q8 did you think of the other person when you received a reply from them?’.

	Very much		A little		Not at all	
	Num.	Standardized residual	Num.	Standardized residual	Num.	Standardized residual
Low–EQ participants	2	-3.06	5	2.59	1	1.26
High–EQ participants	11	3.06	1	-2.59	0	-1.26
p–value	**0.00**		**0.01**		0.21	

**Table 5:** Residual analysis of ‘Q12 compared with before using the robot, did you think more about the other person?’.

	Much more		A little		Less	
	Num.	Standardized residual	Num.	Standardized residual	Num.	Standardized residual
Low–EQ participants	0	-3.30	8	3.30	0	-
High–EQ participants	9	3.30	3	-3.30	0	-
p–value	**0.00**		**0.00**		-	

**Table 6:** Residual analysis of ‘Q15 did you get tired of using the robot?’.

	Often		Neutral		Never	
	Num.	Standardized residual	Num.	Standardized residual	Num.	Standardized residual
Low–EQ participants	1	1.26	4	2.11	3	-2.59
High–EQ participants	0	-1.26	1	-2.11	11	2.59
p–value	0.21		**0.04**		**0.01**	

## Discussion

### The experience of interpersonal communication

Some questionnaire results showed that some participants’ experience of robot-mediated interpersonal communication differed according to their empathy level. This tendency was also confirmed from participants comments during the interviews. For example, high-EQ individuals commented that “When I didn’t get a reply, I thought maybe my partner was busy.”, “When I received the reply, I thought that my partner was doing well.”, and “I imagined the other person in a good way”. Low-EQ individuals commented that “When I received the reply, I thought my partner touched the robot.” and “I could confirm my partner was alive.” These findings suggest that individuals with higher EQ scores tended to be more aware of the other person’s situation and feelings from the robot, whereas those with lower EQ scores tended to express a more factual or objective assessment of the situation rather than trying to understand the other person’s situation. These results imply that empathy influences the interpretation of the awareness information from others expressed by the robot.

In this study, interpersonal communication between remote locations was facilitated by conveying signals through the robot’s movements, such as raising and lowering of the hands. Although a simple touch-based device [19, 37] could provide similar functionality, the robot used here make it easier for individuals with greater empathy to envisage and think about their partner. Previous research supports this assumption. Tanaka et al. [38] investigated the effects of physical embodiment in a robot on remote conversations. The results showed that the presentation of physically embodied motion and the user’s belief that they were talking with a remote operator made the user feel that they were communicating with a remote operator. Thus, the use of a physically embodied robot rather than a device may be more useful in the development of awareness systems that support human-to-human connections.

### Usability

Except for tiredness when using the robot, there were no relationships between EQ scores and robot usability (Q3, Q18, and Q19). Since EQ is a measure that assess people’s empathy level, it is not surprising that there were no significant differences between responses related to usability of the robot according to empathy level. Regarding tiredness of using the robot, the results showed that individuals with higher EQ scores showed less tiredness. This finding may be associated with acceptance of the robot. Previous research has shown that empathy affects the acceptance of technologies, such as social robots [39]. It is possible that individuals with higher EQ scores who were more accepting of using an unfamiliar robot experienced less tiredness when using the robot. However, it is important to note that we cannot provide conclusive evidence to support this suggestion; additional research is needed to confirm this.

### The impact of empathy on the quality of communication

In this study, we conducted a comprehensive evaluation of user empathy without differentiating between cognitive and affective empathy components. The results indicate that empathy plays a substantial role in robot-mediated interpersonal communication. However, it is important to consider that an awareness system that only conveys abstract information, like our robot, may predominantly evoke cognitive empathy and insufficiently evoke affective empathy. When a user receives a signal from a partner through the robot, the user may engage in cognitive empathy by interpreting and predicting the meaning of the signal. However, the simplicity of the signal may limit the user’s ability to respond with appropriate emotions, because the actual intentions, activities and emotions of others are unknown. This may hinder the experience of affective empathy. Given this assumption, it is possible that individuals with lower empathy find it difficult to think about their partners because they lack cognitive empathy.

Regarding the relationship between EQ and interpretation ability, study [40] analyzed the impact of empathy on emotion recognition using both eye-only and whole-face images. The results indicated that there was a positive correlation between EQ and emotion recognition ability. Furthermore, it was found that the ability to recognize emotions decreased when participants were shown eye-only images. In other words, having less information makes it difficult to recognize emotions. Based on these findings, the individuals with lower EQ may show a decline in recognition ability due to its lower empathy and limited information. On the other hand, when more information is provided, a certain level of recognition ability can still be expected for individuals with lower EQ. Therefore, providing additional intuitive information for these low-empathy individuals, such as information about their partner’s emotions or activities, may help them to more easily imagine and understand their partner.

To enhance the quality of interpersonal communication, we provide the design implications that utilize the robot’s posture and eye expressions. By incorporating a mechanism that enables the robot to change its posture to express emotions, it is expected that it will be easier to interpret partner’s emotions. For instance, users can express their positive and negative emotions by manually adjusting the robot’s posture backward and forward. Additionally, the robot’s eye can be utilized to express various user emotions using different shapes and colors. To accomplish this, the robot can be equipped with a camera and emotion recognition software, enabling it to adjust the eye shape and color on an LCD monitor in response to the user’s emotions.

### Limitation

Our research has several limitations that should be considered in future research. First, the findings of this study were obtained from a small sample size of 10 parent-child pairs. To ensure the reliability of the results, further investigation with a larger sample size is required. Second, participants’ empathy was measured without differentiating between cognitive and affective empathy components. From our results, it is assumed that cognitive empathy primarily played a significant role in the interpersonal communication using the robot. To gain a deeper understanding of the relationship between empathy and the experience of the interpersonal communication, the assumption should be validated by measuring cognitive and affective empathy components independently.

## Conclusion

The objective of this study was to comprehensively investigate the effect of empathy on communication mediated by a robot that can convey user’s awareness information. To achieve this goal, we conducted an experiment to examine long-distance communication among distant family members using a robot that can convey their awareness. The experiment, conducted over approximately 1 week with 10 pairs of family members, showed that individuals with higher empathy can better interpret their partner’s situation and feelings from the awareness information. In addition, they tend to feel less burdened in the communication mediated by the robot.
